# HIV and AIDS related knowledge, sources of information, and reported need for further education among dental students in Sudan- a cross sectional study

**DOI:** 10.1186/1471-2458-8-286

**Published:** 2008-08-14

**Authors:** Elwalid Fadul Nasir, Anne Nordrehaug Åstrøm, Jamil David, Rouf Wahab Ali

**Affiliations:** 1 Faculty of dentistry, University of Science and Technology, Sudan; 2 Centre for International Health, University of Bergen, Norway; 3 Institute of Clinical Dentistry, Faculty of Medicine and Odontology, University of Bergen, Norway

## Abstract

**Background:**

Information on the HIV and AIDS-related knowledge among dental students provides a crucial foundation for efforts aimed at developing an appropriate dental curriculum on HIV and AIDS, and for attracting the attention of dental school educators towards the subject.

**Purposes:**

Focusing on a census of dental students attending their 3^rd^, 4^th ^and 5^th ^study year at publicly – and privately funded dental faculties in Khartoum, this study aimed to assess the prevalence and socio-economic correlates of dental students' knowledge, sources of information and reported need for further education related to HIV and AIDS.

**Methods:**

At the time of the survey (March–May 2007), the total number of dental students registered was 782 of which 642 (response rate 82%, mean age 21.7 year, 72% girls) completed anonymous self-administered questionnaires in supervised class room settings.

**Results:**

A total of 49% and 86% had correct sum scores with respect to knowledge of transmission through contamination and through shaking hands and eating, respectively. About half the dental students recognized a need for further education across HIV related issues, varying from 75% (basic HIV/AIDS related issues) to 84% (patient management). Only 38% of the students had correct sum scores regarding various occupational groups at risk for contacting HIV and AIDS. Multiple logistic regression analysis revealed that compared to privately funded dental school students, publicly funded dental school students were less likely to have correct knowledge about modes of HIV transmission (OR = 0.6) and occupational risk groups (OR = 0.6) and to have received information from lectures/health care workers (OR = 0.5).

**Conclusion:**

Students attending privately funded schools were more knowledgeable about various HIV related issues than students from publicly funded schools. About half of the students investigated had received HIV/AIDS information from various sources and reported need for further education. This suggests that students are not adequately prepared for treating patients with HIV infection and AIDS and that the dental school curriculum needs improvements. Differences between public and private dental schools should be considered when planning such improvements.

## Background

Sudan is the largest country in Africa with a prominent diversity in; culture, religion, languages and ethnicity. Being bordered by nine countries, some having a high prevalence of human immunodeficiency virus infection and acquired immunodeficiency syndrome (HIV and AIDS) and having experienced long term ethnical and political conflicts, Sudan is vulnerable for an increase in the prevalence of HIV and AIDS [1]. The first case of HIV and AIDS in Sudan was reported in 1986 and in 2002 the total number of cases reported had increased to 4004 [1]. As in other sub Saharan African countries, the main mode of transmission is heterosexual, accounting for 97% of the HIV positive cases. According to a population based study conducted in 2002, the sero-prevalence was estimated to be 1.6% [1]. Among women attending antenatal clinics, sex workers, prisoners, soldiers, subjects infected with sexually transmitted diseases (STDs) and university students the prevalence of HIV and AIDS has been estimated to vary between 0,5% (soldiers) and 4.4% (sex workers). Moreover, evidence suggests that the HIV and AIDS prevalence among blood donors in Sudan have increased from 0.15% in 1993 to 1.4% in 2000 [1]. Sudan is currently recognised by the World Health Organization, [2] to be a country with an intermediate HIV and AIDS prevalence [1]

It is generally accepted that both dentists and other health care workers should provide quality treatment and care to all individuals indiscriminately [3]. Dentists have a professional and ethical responsibility to provide treatment to patients with HIV and AIDS, particularly since oral lesions, such as hairy leukoplakia, candidiasis and Kaposi's sarcoma, are commonly found in HIV-infected patients [4,5]. Thus, Dental associations (Canadian Dental association, American Dental Association) have issued the recommendations that dentists are obliged to provide quality care for patients with infectious diseases, including HIV and AIDS. Because of the possible transmission of HIV virus through direct contact with blood, the risk for cross-infection comes into particular focus in dental practices [6]. Cross-infection can theoretically take place from patient to patient, from dentist to patient and vice versa. The estimated risk of HIV transmission from general practice dentists to their patients is recognized to be minimal if infection control guidelines are strictly adhered to. About 90% of the HIV infections among health care workers occur in developing countries where occupational safety is a neglected issue [7].

Fear of HIV contagion generates major concerns among health care personnel and may produce a barrier to successful educational efforts about AIDS. This might lead to a variety of adverse outcomes such as unwillingness to treat AIDS patients altogether [8]. Fear about contagion or AIDS phobia have among other things been attributed to lack of proper knowledge about HIV and its transmission routes [9]. It has been suggested that health care workers are deficient in appropriately managing and counseling HIV and AIDS patients and lack sufficient knowledge of symptoms as well as how to diagnose and treat infected patients appropriately [10,11]. A study among medical students in the United States of America revealed that over half of the students investigated believed that treating AIDS patients might be hazardous and that their education had not prepared them to treat those patients safely [8]. In a study from United Kingdom, dentists demonstrated good knowledge regarding oral lesions associated with HIV and AIDS, but were less familiar with HIV and AIDS transmission routes [12]. Among Tanzanian health care workers inadequate knowledge of treatment procedures related to HIV and AIDS caused reluctance with respect to providing care for HIV-positive patients [13]. Few medical students in Pakistan were found to have accurate knowledge regarding symptoms of HIV and AIDS and modes of HIV transmission, while most of them expressed a need for further education [11]. Similar findings have been reported among Turkish nurses and dentists in Kenya, Brazil, Singapore, South Africa and Iran [14-18]. In Sudan, however, only limited information is available in this area.

## Purpose

One major step in preventing a widespread HIV-infection and AIDS epidemic in Sudan is to make certain that dentists are prepared to recognize and treat HIV infected persons and to counsel patients about risks that might lead to infection. Valid knowledge about HIV transmission routes and the HIV epidemiology is important in light of the increasing epidemic and since students are in a process of developing into their subsequent role as dentists in clinical practices. Dental students in Sudan are trained in the epidemiology; the microbiology and pathology (agent, immunology, course and manifestation); and precaution and prevention of HIV during their basic years (1st and 2nd years). In the 3rd, 4th and 5th years students' encounter with HIV patients becomes more apparent. It is upon this background that the present study set out to assess HIV and AIDS related knowledge, sources of HIV and AIDS related information, and perceived need of further education in dental students attending public and private dental schools in Khartoum. Information regarding dental students' HIV-related knowledge provides a crucial foundation for efforts aimed at developing a curriculum that provides clear and consistent information regarding HIV and AIDS and might attract dental school educators' attention to the subject of HIV and AIDS in Sudan.

## Methods

### Study participants

The present cross-sectional study was carried out in April–May 2007 among a census of dental students attending the 3^rd^, 4^th ^and 5^th ^study year in 6 dental faculties in Khartoum, the capital of Sudan. A list of all the dental faculties was obtained from the Ministry of Higher Education and lists of all registered students in the 3^rd^, 4^th ^and 5^th ^years were obtained from all faculties through the Dean's office. The faculties included in this study were publicly and privately funded. Moreover, they represent all available dental faculties in Sudan admitting clinical students and thus include students from all over the country. The total number of dental students registered by the time of the survey was 782 (3^rd^, 4^th ^and 5^th ^graders) out of which 642 students (response rate 82%, mean age 21.7, 82% girls) completed self-administered, anonymous questionnaires in supervised (by teaching assistants) class-room settings. The main reason for non-participation was absenteeism on the day of the data collection.

Written informed consent was obtained from all participants. A formal ethics waiver was received from the research committee at the University of Science and Technology. All included faculties provided approval letters before conducting the study. Before being used in the field, the questionnaire was reviewed by experienced local researchers, dental academics and health administrators.

### Measures

The survey instrument, constructed and administered in English, contained 44 questions regarding HIV and AIDS related knowledge, sources of information and need for further education of dental students. This instrument has been employed previously among university students in East Africa [19].*Sources of HIV and AIDS related information *were assessed using 6 questions, "How much information about HIV and AIDS related issues have you received from-1) *lectures-, 2) radio/TV-,3) internet-,4) reading materials-, 5) friends/relatives-,6) health care workers*". Each question had response categories ranging from (1) "very much" to (5) "non at all". For cross tabulation each question was dichotomized into (0) "some/little information received" (original categories 3, 4, 5) and (1) "much/very much information received" (original categories 1, 2). Two formative sum scores were constructed yielding *"Amount of information received from reading materials/media" *(information received from Radio/TV, internet, reading materials, friends/relatives) and *"Amount of information received from lectures/health care workers" *(information received from lectures, health workers). For cross tabulation and logistic regression analysis the sum scores were dichotomized based on a median split yielding (1) "very much/much information received from reading materials/media and health care workers" and (0) "some/little/none information received from reading materials/media and health care workers".

#### Need for further education

6 questions were applied *in terms of*. "How do you rate your need for further education regarding 1) basic HIV/AIDS related topics, 2) patient management, 3) treatment recommendations, 4) advice on referrals, 5) clinical manifestations, 6) psycho-social complications". Each question had a response category in the range (1) "no need" to (4) "great need". For cross tabulation analysis each question was dichotomized into (1) "moderate/great need" (original categories 3, 4) and (0) "no need" (original categories 1, 2). A formative sum score was constructed based on the 6 questions and labelled *"Need for further information on HIV and AIDS"*. The sum score was dichotomized based on a median split into (1) "moderate to great need" and (0) "slight need/no need" for use in cross tabulation and logistic regression analysis.

#### Knowledge about modes of HIV and AIDS transmission was assessed using the statements

"HIV and AIDS can be transmitted by 1) using contaminated sharp instruments-, 2)unsafe blood transfusion, 3) contaminated blood transfusion, 4) not transmitted through shaking hands-, 5) not transmitted through eating with infected people". In addition, the students replied to the statement "Condom use is safe prevention". Each statement had response category in the range from (1) "strongly agree" to (5) "strongly disagree". For cross-tabulation each statement was dichotomized yielding (1) = "correct knowledge" (original categories 1, 2) and (0) "incorrect knowledge" (original categories 3, 4, 5). Two sum scores were constructed from 5 variables yielding *"Transmission through contaminated instruments*"(unsafe blood transfusion, contaminated blood transfusion, contaminated sharp instruments) and *"Transmission through shaking hands and eating" *(shaking hands, eating). in addition to the one item assessing "Condoms are safe prevention". The two sum scores were dichotomized on a median split into (1) = "Correct overall knowledge" and (0) "Incorrect overall knowledge".

*Knowledge of risk groups for HIV and AIDS*, was assessed using 6 statements with response scale (1) "correct", (0) "incorrect". The students were asked "Is it correct or incorrect that university students, barbers, truck drivers, soldiers, teachers and health care workers are considered as high risk groups by profession"? One formative sum score was constructed from the 6 variables. For logistic regression analysis the sum score was dichotomized based on a median split into (1) "Correct knowledge on occupational risk groups", (0) "Incorrect knowledge on occupational risk groups"

### Statistical procedure

Data were analyzed using the Statistical Packages for Social Sciences (SPSS), version 15. Differences between students from public and private institutions were analyzed using cross-tabulation and Pearson's chi-squared test. Multivariate analysis was conducted by multiple logistic regression analysis with odds ratio (OR) and 95% Confidence Interval (CI). P-value for statistical significance was set to 0.05.

## Results

### Characteristics of participants

A total of 642 students (mean age 21.7 yr, 72% females) 48.6% attending publicly funded and 51.4% attending privately funded dental schools in Khartoum (3^rd^, 4^th^, 5^th ^year of study) participated in the study. The response rate was 82%. A total of 32.7% and 23.1% (p < 0.001) of students attending respectively, private and public dental schools were males, whereas 42.2% of public dental school students and 59.1% of the private dental school students were in the older age group of 22–30 year (p < 0.001). Compared to the public dental schools, the private schools had a higher proportion of students who were smokers and who lived outside Sudan (p < 0.05). Table 1 gives the percentage distribution of participants' socio-demographic characteristics in public and private dental schools in Khartoum.

**Table 1:** Sample profile: Percentage distribution (n) of students' socio-demographic characteristics in public and private educational institutions.

	**Public university**	**Private university**	**Total**
	**% (n)**	**% (n)**	**% (n)**
Female	76.9 (240)	67.3 (216)**	72.0 (456)
Male	23.1 (72)	32.7 (105)*	28.0 (177)
18–21 yr	57.8 (178)	40.8 (131)	49.1 (309)
22–30 yr	42.2 (130)	59.2(190)**	50.9 (320)
Father's education-low	21.7 (67)	16.0 (52)	18.8 (119)
Father's education-high	77.6 (242)	82.7 (273)	80.2 (515)
Mother's education – low	56.5 (174)	49.5 (160)	52.9 (334)
Mother's education- high	45.6 (134)	51.6 (163)	48.7 (297)
Smoking ever- no	92.6 (289)	79.5 (260)	85.9 (549)
Smoking ever -yes	7.4 (23)	20.5 (67)**	14.1 (90)
Living in Khartoum	58.0 (181)	45.9 (151)	51.8 (332)
Living outside Sudan	42.0 (131)	54.1 (178)*	48.2 (309)

** p < 0.001

* p < 0.05

### HIV and AIDS information received from various sources

Lectures and Radio/TV were the most frequently reported sources of information related to HIV and AIDS as reported by 61% and 44% of the students, whereas information from friends/relatives (31%) and health care workers (39%) were less frequently reported. As shown in Table 2, public dental students reported more frequently to have received information from Radio/TV (63% versus 44%), whereas private dental students reported more frequently than public dental students to have received information from lectures (67% versus 53%). Private dental students reported more often than their public dental student counterparts that they had received much and very much information from lectures/health care workers (63% versus 44.6%, p < 0.001) (Table 2).

**Table 2:** HIV/AIDS related information received from various sources and need for further education by type of university.

***Sources of information***	**Public university**	**Private university**	**Total**
	**% (n)**	**% (n)**	**% (n)**
Lectures	53.8 (168)	67.8 (221)**	61.0 (389)
Radio/TV	50.0 (156)	38.4 (126)*	44.1 (282)
Internet	28.6 (89)	36.6 (119)*	32.7 (208)
Reading materials	34.5 (107)	35.6 (116)	35.1 (223)
Friends/relatives	29.8 (93)	32.8 (107)	31.3 (200)
Health care workers	33.3 (104)	45.1 (147)*	39.3 (251)
Sum score: Information from media/reading	54.2 (165)	59.4 (193	56.4 (358)
Sum score: Information from lectures/health care workers	44.6 (139)	63.0 (204)**	53.9 (323)
***Need for further education***			
Basic HIV/AIDS issues	81.1 (249)	70.9 (232)*	75.9 (481)
Patient management	88.1 (274)	80.4 (263)*	84.2 (537)
Treatment recommendations	85.3 (262)	74.8 (243)*	79.9 (505)
Advice on referrals	81.2 (250)	72.4 (236)*	76.7 (486)
Clinical manifestations	86.1 (267)	77.9 (254)*	81.9 (521)
Psycho-social complications	83.2 (258)	78.2 (254)	80.6 (512)
Sum score: need for further information-	48.2 (145)	47.0 (151)	47.6 (296)

Percentages (n) of those who confirmed much/very much information received and moderate/great need for further information.

** p < 0.001, * p < 0.05

### Perceived need for further education

About half of the students, 47.6%, confirmed a moderate to high need for further education across topics related to HIV and AIDS (Table 2). Substantial proportions of students wanted to know more about the management of HIV infected patients (84%), clinical manifestations (81%), psycho social complications (80%), treatment recommendations (79%) and basic HIV and AIDS issues (75%). Statistically significantly higher proportions of students from public dental schools than from private dental schools reported need for further education regarding basic HIV and AIDS issues, patient management, treatment recommendations, advice referrals and clinical manifestations (p < 0.05) (Table 2).

### Knowledge regarding people at risk for HIV and AIDS

Substantial proportions of students from both public- and private dental schools classified correctly across various occupational groups at risk for getting HIV -infection (38.5%) (Table 3). Health workers (79.1%) and barbers (74.5%) were the risk groups most frequently recognized, whereas teachers (10.7%) and truck drivers (35.9%) were less frequently recognized as risk groups in this context. Knowledge about risk groups varied across institutions with higher proportions of private dental school students than public dental school students classifying correctly university students, truck drivers and teachers as occupational risk groups.

**Table 3:** Knowledge about HIV related issues versus type of university. Percentage (n) of those who replied correctly to the various items

	**Public**	**Private**	**Total**
	**% (n)**	**% (n)**	**% (n)**
***Risk groups***			
University students	48.9 (152)	64.2 (210)**	56.7 (362)
Berbers	75.5 (234)	73.5 (239)	74.5 (473)
Truck drivers	24.8 (77)	46.5 (152)**	35.9 (229)
Soldiers	64.2 (199)	63.5 (207)	63.8 (406)
Teachers	6.4 (20)	14.7 (48)*	10.7 (68)
Health workers	87.4 (271) **	71.3 (233)	79.1 (504)
Sum score: Knowledge of risk groups	30.7 (95)	46.0 (149)**	38.5 (244)
***Modes of transmission***			
Condom safe prevention	46.9 (146)	53.0 (174)	50.1 (320)
HIV/AIDS transmits by unsafe blood transfusion	95.8 (299)	96.3 (316)	96.1 (615)
HIV/AIDS transmit by contaminated blood transfusion	97.4 (304)	97.0 (318)	97.2 (622)
------ transmits by contaminated sharp instruments	93.9 (293)	93.9 (307)	93.9 (600)
----do not transmit by shaking hands with infected persons	88.1 (275)	83.1 (271)	85.6 (546)
--------do not transmit by eating with infected persons	90.1 (281)	85.6 (280)	87.8 (561)
Sum score: Transmission through contaminated instruments	43.9 (137)	53.8 (176)*	49.0 (313)
Sum score: Transmission through shaking hands/eating	43.6 (278)	43.5 (275)	86.6 (553)

** p < 0.001

* p < 0.05

### Knowledge of HIV transmission modes

A majority of students had accurate knowledge regarding modes of transmission with the proportions answering each question correctly varying between 85% (do not transmit by shaking hands) and 97% (transmission through contaminated blood transfusion). About half the students recognized condom use as a safe method of preventing transmission of HIV. Knowledge of modes of transmission did not vary statistically significantly differ between students attending public- and private dental schools. Good overall knowledge about transmission through contaminated instruments (sum score) was more frequently reported by private dental school students than by their public counterparts (53.8% versus 43.9%, p < 0.05) (Table 3).

### Socio-demographic correlates of indicators related to information and knowledge of HIV/AIDS

To control for possible effect of confounding, all sum scores were regressed on several socio-demographic factors in terms of type of educational institution, gender, age, place of living and parents' education. As shown in Table 4, compared to being a private dental school student and having a father with high education, public dental school students and students having fathers with low education had reduced odds (OR = 0.6) for reporting good knowledge regarding HIV transmission through contaminated instruments. Public dental school students and younger age groups were less likely than their counterparts in the opposite groups to report information received from health care workers/lectures (OR = 0.5) and to have good knowledge on HIV and AIDS related occupational risk groups (OR = 0.6). Sum scores regarding information received from media/reading material, perceived need for further information, and HIV and AIDS transmission through handshaking and eating did not vary statistically significantly with any of the socio-demographic characteristics measured in this study.

**Table 4:** Socio-demographic factors associated with knowledge of transmission, amount of information received and knowledge on occupational risk groups Odds ratios (OR) and 95% confidence interval (CI).

	**Transmission contaminated instruments**	**Information received/health workers/lectures**	**Occupational risk groups**
	OR (95% CI)	OR (95% CI)	OR (95% CI)
Private institution	1	1	1
Public institution	0.6 (0.4–0.9)	0.5 (0.3–0.7)	0.6 (0.4–0.8)
Male	1	1	1
Female	1.2 (0.8–1.7)	1.2 (0.8–1.7)	1.0 (0.7–1.5)
22–30 yr (older)	1	1	1
18–21 yr (younger)	1.1 (0.7–1.5)	0.6 (0.4–0.8)	0.6 (0.4–0.8)
Father's education – high	1	1	1
Father's education low	0.6 (0.4–0.9)	1.1 (0.7–1.7)	0.7 (0.4–1.1)
Mother's education- high	1	1	1
Mother's education – low	1.5 (1.1–2.1)	1.0 (0.7–1.4)	0.9 (0.6–1.3)
Living in Khartoum	1	1	1
Living outside Sudan	1.3 (0.9–1.8)	0.8 (0.6–1.2)	0.7 (0.5–1.0)

## Discussion

The present study is the first to assess detailed knowledge regarding the modes of transmission of HIV-infection, related aspects of infection control, sources of HIV information and perceived need for further education regarding HIV and AIDS among Sudanese dental students. The findings are believed to be representative for dental students attending 3^rd^, 4^th ^and 5^th ^study year in Sudan since a census of available dental schools in Sudan was considered with a high response rate. The study group targeted represents a restricted range of adults regarding age and socio-economic background making it less probably that social inequalities have contributed largely to the pattern of results. On the other hand, universities might vary regarding their format of instructions and in their atmosphere. Thus, universities vary regarding the type of students they attract. This was apparent in that public dental schools tended to have more female students and fewer smokers than the private dental schools. In addition, private dental schools encompassed older students living outside Sudan probably reflecting the higher socioeconomic status necessary of being in the position to attend private universities. In accordance with the demography of all higher educational institutions in Sudan, a larger proportion of females than males participated in the present study, reflecting the general progress in girls' education going on in this country.

As shown in Table 3, the present results suggest that dental students who should be aware of the latest information regarding HIV and AIDS demonstrated moderate to good overall knowledge with proportions having correct sum scores on occupational risk groups, transmission through contamination and transmission through shaking hand and eating ranging from 38% to 86%. Moreover, the percentages having correct knowledge varied from 35% (truck drivers as occupational risk groups) to 97% (transmission through contaminated blood transfusion). These figures show little difference from those obtained regarding the knowledge level in the general population of 15–24-year-olds from various countries, suggesting that about 50% have serious misconceptions regarding HIV transmission. Lack of knowledge was also apparent among the Sudanese dental students in that only 10% and 35% of students correctly classified teachers and truck drivers as occupational risk groups. Similar proportions of subjects having misconceptions with respect to HIV related issues have been identified in previous studies from industrialized as well as non-industrialized countries [12,15,17,18,20,21]. On the other hand, Brook [22] found relatively sufficient knowledge about transmission routes and high-risk group among Israeli high school students. It should be noted that knowledge of the risk of being infected with HIV attributed to occupational groups, such as teachers and health care workers are seldom certain. Thus, there is disagreement among experts as to the relative importance of those groups when it comes to their susceptibility for infection. Such disagreement might explain differences in terms of private school students rating the vulnerability of university students, truck drivers and teachers higher than did their public dental student counterparts, whereas public dental school students rated the vulnerability of health care workers higher than did private dental school students.

The observation that a substantial proportion of the participants (79%) were concerned about the risk status of health care workers might reflect a fear of contagion among dental students. Previous studies have suggested that between 26% and 90% of students of medical subjects are concerned about HIV transmission from patients [8]. It is evident that students' beliefs that working with AIDS patients endangers their health might develop into an unwillingness to treat AIDS patients altogether [23]. Generally, knowledge about HIV infection is recognized as a critical component for the control and management of this disease [24]. Several authors have attributed fear of contagion to inadequate HIV and AIDS related knowledge, whereas others have demonstrated a reduction in fears through improved AIDS education [11,25]. Apparently, there is a need for better professional education concerning HIV and AIDS among health care workers throughout the world. Proper training in medical and psychological aspects of treating HIV positive patients has been recognized as an effective method to change the attitudes towards HIV and AIDS among dental health care workers [25,26]. Evaluations of public educational programs in developed- and developing countries dealing with prejudice reveal that the media images of the epidemic gradually become more positive and informative leading to a gradual shift in attitudes among health care workers.

Although a majority of the Sudanese dental students were aware of the common transmission routes for HIV infection, their knowledge was less accurate with respect to other HIV- and AIDS related topics. About half of the students investigated reported need for continued education considering HIV and AIDS and admitted having received much information on from media/reading material and lectures/health care workers (Table 2). The consistency of the present findings suggest that Sudanese dental students might be less well prepared for the task of treating patients with HIV infection and AIDS and that the dental school curriculum regarding HIV and AIDS needs some improvements. The finding that between 75% (basic HIV related issues) and 81% (clinical manifestations and psycho-social complications) of the participants reported a need for further education also suggests a recognition made by them of AIDS being a threat to the Sudanese society. These findings are similar to those reported by American and European investigators one decade ago when the AIDS epidemic was emerging [27]. Consistent findings have also been reported previously in studies from developed and developing countries [17,18,23,28]. A recent study of dentists in Kenya revealed that the majority reported having a need for further HIV and AIDS education [16].

Lectures (61%), media (44%) and health care workers (39%) were the most frequently reported sources of HIV information among both public and private dental students. The sources of AIDS information reported in this study are similar to those identified previously among dental health care workers from Europe, USA, Japan and Iran, as well as in students of medical subjects from Iran and Pakistan [14,26]. The present results are in contrast to a study of Japanese dental health care workers where TV and newspapers were cited as the most common source of AIDS related knowledge [26]. Sudanese dental students rating TV and Radio as a less common AIDS informational sources this has to be viewed in light of how this topic has been evaluated by the Sudanese national media. Recognized as sensitive sexually related issues, Sudanese media has not yet been in favor of public educational campaigns considering HIV and AIDS [29]. Dental health care workers in Netherlands and Italy cited scientific journals and continuing educational courses as their most important source of HIV related information. In contrast, Sudanese dental students reported dental reading materials as a relatively less common source of information thus highlighting a deficiency in their current education curricula concerning HIV and AIDS.

Analyzing all sum scores of HIV-related knowledge according to demographic covariates in multiple logistic regression analyses made it possible to control for confounding influences and to identify the independent effect of each socio-demographic factor. Notably, both in univariate- and multivariate analyses there was a consistent trend of public dental students showing less accurate knowledge about HIV and AIDS related issues than their private dental student counterparts. This reflects the existence of a social gradient among students attending different parts of the Sudanese educational system. Compared to public dental students, private students were mainly older, included lower proportions of women and higher proportions of subjects that were living outside Sudan indicating the higher socio-economic status needed to be able to attend privately funded and governed universities. As compared to students attending private institutions and having father's of high education, students from public dental institutions and those having father's with low education were less likely to be knowledgeable about modes of HIV transmission. Moreover, public dental students tended to be less likely to have received information from health workers and lectures and to be knowledgeable about risk groups. These differences might reflect variations in the HIV related attitudes and behaviors of the private- and public dental faculties as well as variation in the amount of material, lectures and number of clinical encounters regarding HIV and AIDS that are offered to students. The trend of younger students having less accurate knowledge than their older counterparts across HIV related issues corroborate previous findings among Pakistani medical students and suggests that students' knowledge level improves as they progress through their education programs [11].

The results of the present study should be interpreted with caution since it is limited by the use of self-administered questionnaires and not by interviews which are most commonly used in non-occidental settings and by the inclusion of students volunteering to participate. It might be argued, however, that anonymous self reports is more reliable than interview data in that the respondents are less prone to the pressures of social demand emanating from conversation face to face with a research assistant [30]. Moreover, the questions employed were sufficiently simple and unambiguous to achieve a reasonable degree of validity on the different variables. In fact previous researches have found such self- reports to be quite reliable.

## Conclusion

The results of the present study revealed that Sudanese dental students have deficiencies with respect to their knowledge and amount of information received on HIV and AIDS related topics. This deficiency was more frequently observed in public dental students than in their private dental student counterparts. The results indicate that dental students in Sudan are less well prepared for their future task of treating patients with HIV infection and AIDS and highlight a need for improving the dental school curriculum regarding HIV and AIDS. Dental school based differences in terms of privately and publicly funded schools should be considered when planning improvements in the HIV and AIDS curriculum. Thus, the present findings might serve as a wake up call for dental program directors to explore further how well graduating students in the different institutions are prepared to treat patients at risk of or infected with HIV and AIDS. Dental students' interest in this topic, as revealed by their recognition of a need for further education reflects a susceptibility for change that should be both supportive and encouraging for dental educators in their efforts towards improving dental education on HIV and AIDS related topics in Sudan

## Competing interests

The authors declare that they have no competing interests.

## Authors' contributions

EFN: principle investigator, conceived of the study, designed the study, collected data, performed statistical analyses and manuscript writing. ANÅ: main supervisor, guided the design of the study and has been actively involved in statistical analyses of data and in manuscript writing. JD: co-supervisor, has been actively involved in statistical analyses and paper writing. RWA: co-supervisor, has provided valuable comments on the paper in general and has been actively involved in manuscript writing. All authors read and approved the final manuscript.

## Pre-publication history

The pre-publication history for this paper can be accessed here:


